# Effect of triple therapy in patients with asthma-COPD overlap

**DOI:** 10.5414/CP203382

**Published:** 2019-06-24

**Authors:** Yoshihisa Ishiura, Masaki Fujimura, Noriyuki Ohkura, Johsuke Hara, Kazuo Kasahara, Nobuyasu Ishii, Takeshi Tamaki, Toshiki Shimizu, Shosaku Nomura

**Affiliations:** 1 First Department of Internal Medicine, Kansai Medical University, Osaka,; 2 Respiratory Medicine, Toyama City Hospital, Toyama,; 3 Respiratory Medicine, National Hospital Organization Nanao Hospital, Nanao, and; 4 Respiratory Medicine, Kanazawa University Hospital, Kanazawa, Japan

**Keywords:** ACO, asthma, COPD, UMEC, FF/VI, triple therapy

## Abstract

Objective: Asthma-chronic obstructive pulmonary disease (COPD) overlap (ACO) is of increasing interest because ACO patients have significantly worse outcomes, leading to greater social and economic burdens compared with asthma or COPD alone. Some guidelines for ACO recommend triple therapy with inhaled corticosteroids, long-acting β2 agonists, and long-acting muscarinic antagonists. However, this approach is based on extrapolating data from patients with asthma or COPD alone. Therapeutic studies for ACO have not previously been conducted. Materials and methods: A 12-week, randomized, open-label cross-over pilot study was conducted in 17 ACO patients to evaluate the effect of umeclidinium (UMEC) 62.5 µg once-daily added to fluticasone furoate/vilanterol (FF/VI) 200/25 µg once-daily. A 4-week run-in, a first and a second 4-week treatment period were included. Respiratory function, respiratory impedance, fractional exhaled nitric oxide, COPD assessment test, and asthma control test scores were evaluated 0, 4, and 8 weeks after randomization. Results: Mean values of post-bronchodilator forced expiratory volume in 1 second as a percentage of the predicted value (%FEV_1_), after UMEC was added to FF/VI, were significantly higher than after the run-in (p < 0.01). Mean values of resonant frequency during inspiration (Fres), after UMEC was added to FF/VI, were significantly lower than after the run-in (p < 0.01). Conclusion: Adding UMEC to FF/VI provides greater improvement in lung function, indicating that triple therapy is a suitable regular treatment for ACO.

**What is known about this subject**

- Adding UMEC to FF/VI provides improvement in lung function in patients with ACO.

**What this study adds**

- Triple therapy with ICS, LABA, and LAMA is effective on patients with ACO.

## Introduction 

Asthma-chronic obstructive pulmonary disease (COPD) overlap (ACO) has been the focus of attention [1, 2, 3] because patients with ACO have worse health-related quality of life, more rapid disease progression [4], more frequent respiratory exacerbation [5], increased comorbidities, and greater health care utilization, leading to a greater socioeconomic burden than for patients with asthma or COPD alone [6, 7, 8]. ACO is important for general physicians as well as pulmonologists because it is a frequently-encountered clinical entity, with between 15% and 20% prevalence in populations with airway diseases [3, 9]. Triple therapy with inhaled corticosteroids (ICS), long-acting β2 agonists (LABA), and long-acting muscarinic antagonists (LAMA) has lately attracted considerable attention because it has been shown to be a useful and convenient treatment in patients with obstructive airway diseases [10, 11, 12]. Some guidelines also recommend triple therapy with ICS, LABA, and LAMA to improve lung function and respiratory symptoms and to reduce respiratory exacerbations [3, 13, 14]. However, this treatment approach is based on the extrapolation of data derived from studies of patients with asthma or COPD alone because therapeutic studies for ACO have not previously been conducted. Therefore, we conducted this pilot study to compare the efficacy of LAMA/ICS/LABA triple therapy with ICS/LABA dual therapy as a first clinical trial for ACO. 

## Materials and methods 

This was a 12-week, randomized, open-label cross-over pilot study to evaluate the effect of umeclidinium (UMEC) 62.5 µg once-daily via the Ellipta^TM^ dry powder inhaler (GlaxoSmithKline, Ware, UK) added to fluticasone furoate/vilanterol (FF/VI) 200/25 µg once-daily in the morning, also via the Ellipta^TM^ dry powder inhaler. Randomization was carried out by the sealed envelope method. The study was conducted between April 2016 and November 2016. This study (approval no. UMIN000021086) was conducted in accordance with the principles of the Declaration of Helsinki and was approved by the Ethics Committee of Toyama City Hospital. Written informed consent was obtained from all subjects before participating in the study. 

### Subjects 

A total of 17 patients with stable ACO (17 males and no females) with a mean age of 70.1 ± 9.0 years (± standard deviation, SD; range 54 – 87 years) participated in this pilot study. All patients were ex-smokers with a smoking history of 66.6 ± 39.5 pack-years (± SD). We consider that the imbalance in gender may be the result in the habit of smoking in Japan, since smokers among the elderly tend to be male in our country. Each patient was diagnosed with ACO in accordance with past studies conducted by Gibson and others [1, 2, 15, 16, 17]. They had episodic respiratory symptoms, increased airflow variability (asthma: airway hyper-responsiveness (AHR) or bronchodilator response (BDR)) as well as incompletely reversible airway obstruction (COPD: post-bronchodilator forced expiratory volume in 1 second (FEV_1_)/forced vital capacity (FVC) < 70%, and post-bronchodilator FEV_1_ < 80% of predicted values). AHR was defined if there was a 20% FEV_1_ fall from baseline after inhalation of 8 mg/mL or less of methacholine. BDR was defined as an increase in post-bronchodilator FEV_1_ of 200 mL and 12% compared with pre-bronchodilator FEV_1_. 

All patients were allowed to take oral theophylline, leukotriene receptor antagonist, and mucolytic agents, as shown in Table 1. They had not received oral steroid therapy for at least 8 weeks. The study was carried out when patients’ symptoms were mild and stable. 

### Study protocol 

**Figure 1.**
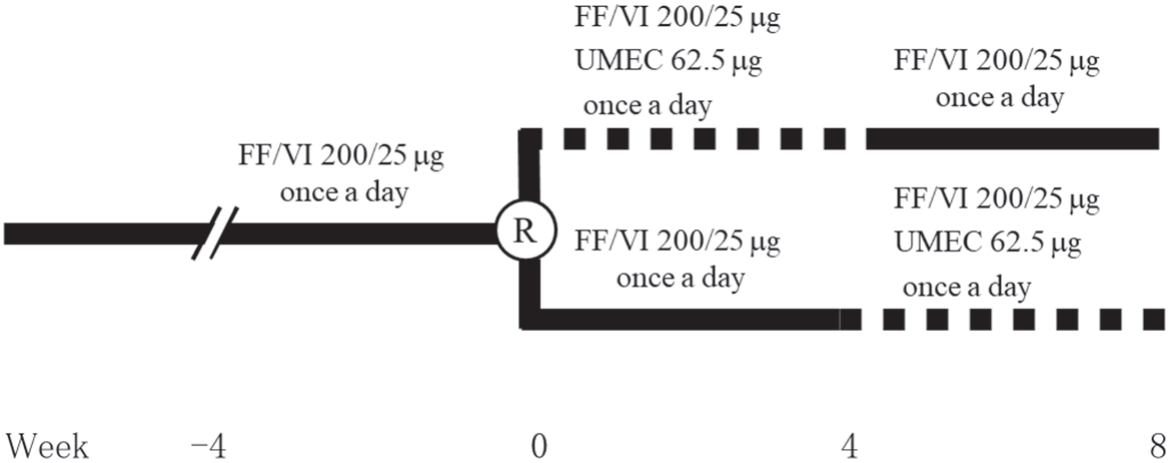
Design for the randomized, open-label cross-over study. Solid line, treatment with fluticasone furoate/vilanterol (FF/VI); dotted line, triple therapy with umeclidinium (UMEC) added to FF/VI. ACT = asthma control test; CAT = chronic obstructive pulmonary disease assessment test; FeNO = fractional exhaled nitric oxide; FOT = forced oscillation technique; R = randomization.

The medication for ACO was stopped at 9:00 PM 2 days beforehand to allow a washout period of 24 hours or more before the measurement of respiratory functions at 10:00 AM on each test day. Each patient attended 4 times, separated by 4 weeks, at the same time each day. They had been receiving FF/VI 200/25 µg once-daily in the morning for at least 4 weeks before randomization, as shown in Figure 1. Subsequently, they were randomized into two groups and underwent treatment with UMEC 62.5 µg once-daily in the morning added to FF/VI 200/25 µg once-daily in the morning, or with FF/VI 200/25 µg once-daily in the morning alone. Measurements of respiratory functions were carried out 0, 4 and 8 weeks after randomization, including vital capacity, FVC, FEV_1_, FEV_1_/FVC, peak expiratory flow, forced expiratory flow at 25 – 75%, maximum expiratory flow rate at 50% forced vital capacity, maximum expiratory flow rate at 25% forced vital capacity, respiratory impedance measured by the forced oscillation technique (FOT), fractional exhaled nitric oxide (FeNO), COPD assessment test (CAT) scores, asthma control test (ACT) scores, electrocardiogram, and blood examinations including peripheral blood eosinophils and immunoglobulin E (IgE). 

### Measurements 

Respiratory functions were measured using a dry wedge spirometer (Chestac 8900^TM^, Chest Co., Ltd., Tokyo, Japan) to assess the bronchodilatory effect for small airway obstruction of the treatment regimens, in the same manner as previously reported [18, 19, 20, 21]. Respiratory impedance was measured by FOT using another device (MostGraph-01^TM^, Chest Co., Ltd.) in accordance with the previously reported recommended techniques [20, 21, 22, 23]. The FeNO level, a surrogate eosinophilic airway inflammatory marker, was measured using a commercially-available device (NIOX MINO^TM^, Aerocrine, Stockholm, Sweden) before any forced expiratory maneuvers [24]. To assess and quantify the effect of COPD symptoms on patient health status, patients were asked to complete a CAT, a simple questionnaire that is a reliable and valid tool to examine the impact of COPD symptoms over time [25]. It comprises 8 items scored from 0 to 5 to give a maximum total score of 40. CAT scores of 1 – 10, 11 – 20, 21 – 30, and 31 – 40, respectively, represent categories of mild, moderate, severe, and very severe health status impairment [25, 26]. To evaluate asthma control status during the previous 4 weeks, patients were asked to complete an ACT, an easy five-question test that evaluates their asthma symptoms [27, 28]. Each question is scored from 1 to 5, giving a total score in the range of 5 – 25, with low scores corresponding to a high level of symptoms and therefore poor asthma control. All adverse events during the study period were recorded. 

### Data analysis 

Data are shown as the mean ± SD. One-way analysis of variance (ANOVA) was used for analyzing differences between the run-in period, the FF/VI treatment period, and the UMEC plus FF/VI treatment period in respiratory functions, FOT parameters, FeNO levels, CAT scores, ACT scores, heart rates measured by electrocardiogram, and blood examinations including IgE. Values for FEV_1_, FVC, and resonant frequency (Fres) were compared pairwise between each of the run-in periods, the FF/VI treatment period, and the UMEC plus FF/VI treatment period using paired t-tests. A p-value of less than 0.05 was taken as significant. These analyses were performed using the software, StatView 4.5J (Abacus Concepts, Berkeley, CA, USA). 

## Results 

**Figure 2.**
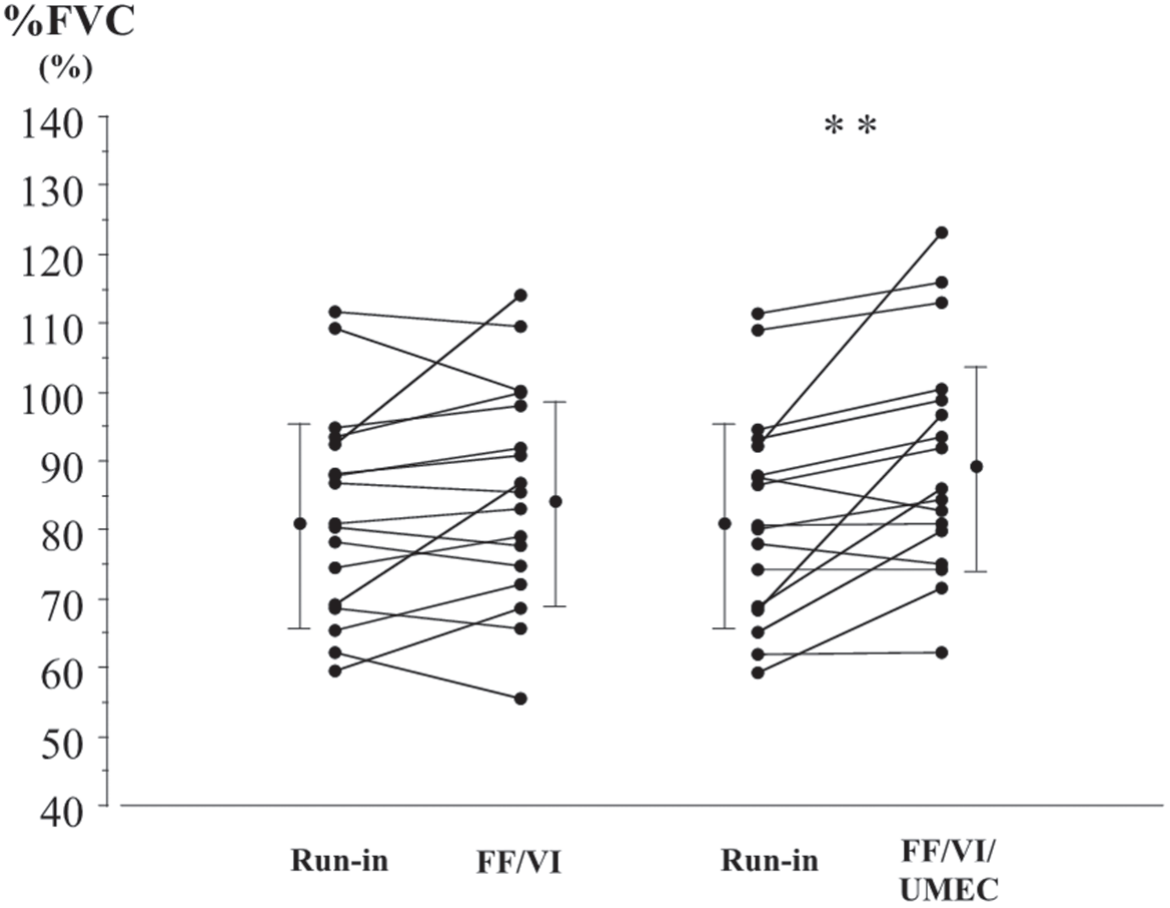
Individual data for forced vital capacity as a percentage of the predicted value (%FVC), before each treatment and after FF/VI and triple therapy with UMEC added to FF/VI, in patients with asthma-chronic obstructive pulmonary disease overlap (ACO). Each panel shows the parameter changes for all patients and the mean ± SD. **p < 0.01 between treatments, determined by paired t-test.

Respiratory functions obtained by spirometry are shown in Table 2. Most parameters were significantly higher after UMEC was added to the FF/VI treatment, compared with the corresponding values after the run-in or the FF/VI treatment period. Changes in FVC as a percentage of the predicted values (%FVC) after the run-in and each treatment period are shown in Figure 2. Mean values for %FVC were 81.5% (± 15.2%) after the run-in, 84.4% (± 15.9%) after the FF/VI treatment period, and 89.2% (± 16.6%) after the UMEC plus FF/VI treatment period. The %FVC values, after UMEC was added to the FF/VI, were significantly higher than those after the run-in (p < 0.01). 

**Figure 3.**
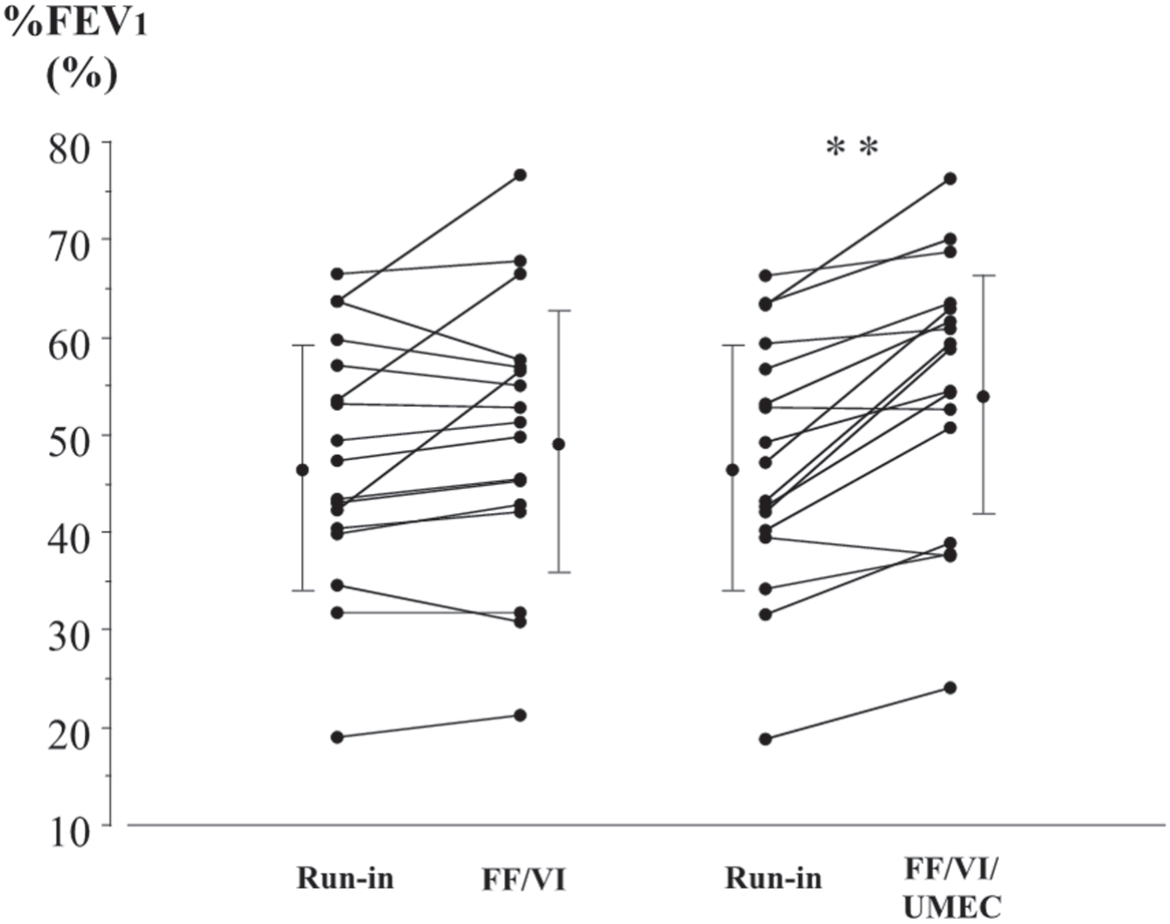
Individual data for forced expiratory volume in 1 second as a percentage of the predicted value (%FEV_1_), before each treatment and after FF/VI and triple therapy with UMEC added to FF/VI, in patients with ACO. Each panel shows the parameter changes for all patients and the mean ± SD. **p < 0.01 between treatments, determined by paired t-test.

Changes in FEV_1_ as a percentage of the predicted value (%FEV_1_) after the run-in and each treatment period are shown in Figure 3. Mean values for %FEV_1_ were 46.8% (± 12.7%) after the run-in, 49.3% (± 14.0%) after the FF/VI treatment period, and 54.4% (± 13.6%) after the UMEC plus FF/VI treatment period. The %FEV_1_ values, after UMEC was added to the FF/VI, were significantly higher than those after the run-in (p < 0.01). Mean values for FEV_1_ were 1.26 L (± 0.42 L) after the run-in, 1.33 L (± 0.51 L) after the FF/VI treatment period, and 1.46 L (± 0.49 L) after the UMEC plus FF/VI treatment period. The FEV_1_ values, after UMEC was added to the FF/VI, were significantly higher than those after the run-in (p < 0.01). 

**Figure 4:**
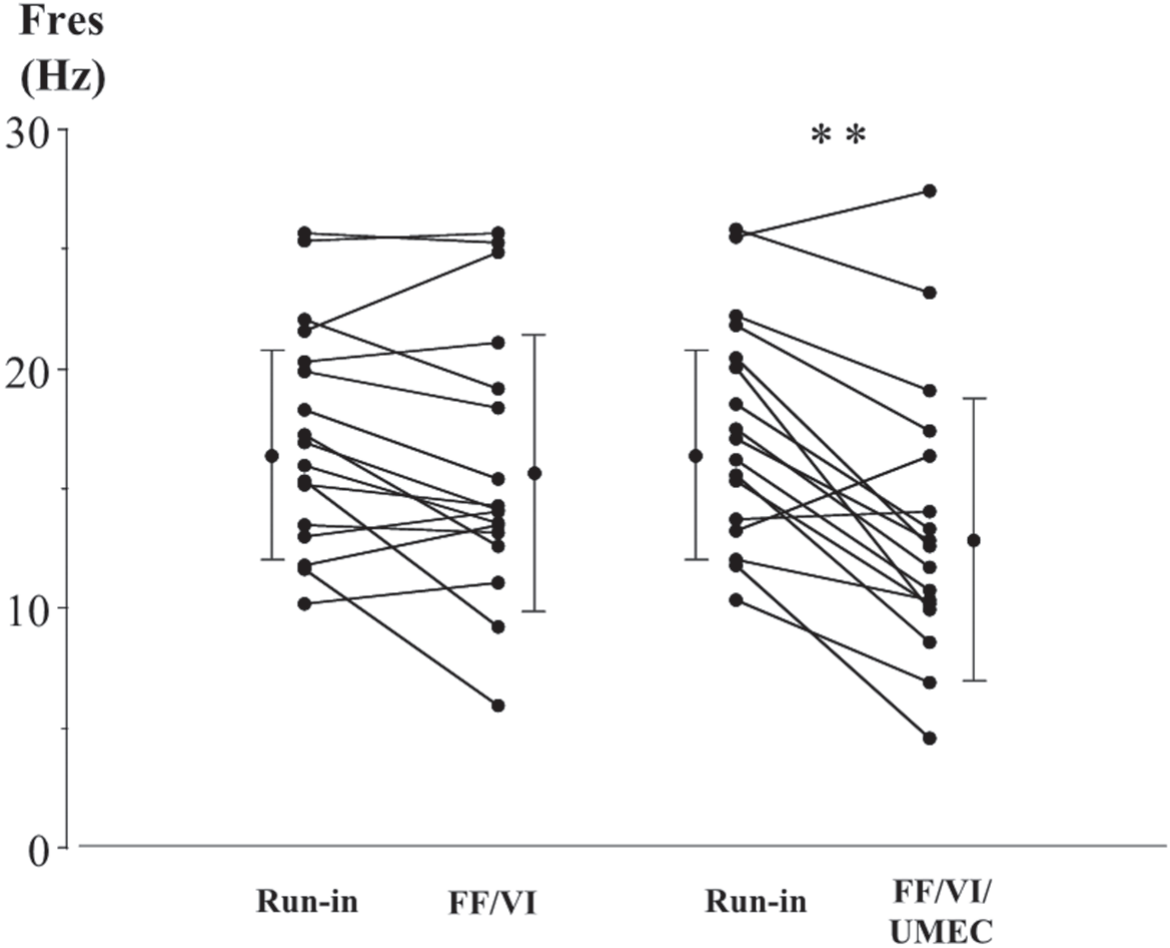
Individual data for resonant frequency during inspiration (Fres), before each treatment and after FF/VI and triple therapy with UMEC added to FF/VI, in patients with ACO. Each panel shows the parameter changes for all patients and the mean ± SD. **p < 0.01 between treatments, determined by paired t-test.

Respiratory impedances during inspiration, as measured by FOT, are shown in Table 3. Each parameter was significantly improved after the UMEC plus FF/VI treatment period compared to after the run-in and the FF/VI treatment period, except for respiratory system resistance at 20 Hz. Fres values during inspiration, after the run-in and each treatment period, are shown in Figure 4. Mean values for Fres were 17.0 (± 4.7) Hz after the run-in, 15.6 (± 5.7) Hz after the FF/VI treatment period, and 13.0 (± 5.8) Hz after the UMEC plus FF/VI treatment period. The Fres values, after UMEC was added to the FF/VI, were significantly lower than those after the run-in (p < 0.01). 

FeNO levels, CAT scores, ACT scores, and blood examinations (eosinophils and IgE) were not significantly different among the run-in, FF/VI treatment, and UMEC plus FF/VI treatment periods, as shown in Table 3. The mean values for heart rate were 67.7 (± 16.8) beats/min. after the run-in, 67.1 (± 17.9) beats/min. after the FF/VI treatment period, and 69.8 (± 19.7) beats/min. after the UMEC plus FF/VI treatment period. Heart rate was not significantly different among the treatment periods. None of the patients enrolled in this pilot study complained of cardiovascular or gastroenterological symptoms after the administration of FF/VI or UMEC plus FF/VI. 

## Discussion 

The study showed that 4-week treatment with UMEC 62.5 µg added to FF/VI 200/25 µg improved lung function and respiratory impedance in patients with ACO, without patients complaining of cardiovascular symptoms. These findings clearly demonstrate that triple therapy with once-daily UMEC added to once-daily FF/VI has potential as a regular treatment for ACO. 

ACO is an increasingly recognized phenotype because it is common in patients with obstructive airway disease with multiple clinical problems [9, 29]. Fu et al. [15] reported that 53.2% of patients with COPD show overlapping asthmatic patterns. Another study group demonstrated a high prevalence of ACO, with 55% of COPD patients presenting an asthma-predominant phenotype [16]. In an UPLIFT trial, nearly 66% of patients with COPD improved their respiratory functions by more than 15% after receiving bronchodilator therapy [30]. These studies show the high prevalence of ACO in clinical medicine. More importantly, previous studies have shown that ACO has a poorer prognosis than asthma or COPD alone [5, 31, 32]. Diaz-Guzman et al. [31] reported that patients with ACO had a higher risk of obstruction on spirometry and death during follow-up. Another study showed that ACO is a stronger determiner of low quality of life than either disease alone [32]. Hardin et al. [5] also reported that patients with ACO had a poorer disease-related quality of life and more severe and frequent COPD exacerbation in the preceding year, with an odds ratio of 3.55. Therefore, patients with ACO utilize a large proportion of available medical resources, resulting in a cost burden as much as two to six times higher than asthma or COPD alone [6]. 

Recently, triple therapy with ICS, LABA, and LAMA has been a focus of interest as a maintenance or a “step-up” treatment from single or double therapy because it has been revealed to be useful, convenient, and to reduce hospitalization rates in patients with obstructive airway diseases [10, 11, 12]. A real-world survey revealed that 29.6% of patients with ACO were prescribed triple therapy as a more aggressive therapy for disease associated with more symptoms and greater physician-perceived risk of exacerbation [33]. The combination of drugs with distinct and complementary mechanisms of action may offer improved efficacy in the treatment of asthma and COPD, which may in turn help to relieve the burden of these diseases on lung function, symptoms, daily activities, and risk of exacerbation [10, 11, 12]. Some guidelines for ACO recommend triple therapy with ICS, LABA, and LAMA to improve lung function and respiratory symptoms and to reduce respiratory exacerbation [3, 13, 14]. However, this treatment approach is based on the extrapolation of data derived from studies of patients with asthma or COPD alone, since randomized clinical trials have not previously been conducted for patients with ACO. We previously reported the efficacy of dual therapy with ICS and LABA as a maintenance therapy for ACO [20]. We conducted the present study as the first clinical trial to investigate the beneficial effect of triple therapy with ICS, LABA, and LAMA, and clearly showed the bronchodilatory effect of this treatment approach for ACO. We cannot explain the precise mechanisms of the beneficial effect because we did not investigate mediators of the sputum. However, we suspect that the anti-inflammatory properties of LAMAs might contribute to this beneficial effect because tiotropium, another LAMA, can control proinflammatory activities in human bronchial epithelial cell lines [34]. 

UMEC, the novel LAMA used as an add-on therapy in this study, has been approved for the maintenance of moderate-to-very-severe COPD symptoms and improves both lung function and health status through the mechanism of blocking acetylcholine-mediated bronchoconstriction by binding to M3 receptors [35]. Several studies have shown the efficacy of UMEC added to FF/VI in patients with COPD [11, 36]. Other studies have also shown that triple therapy including tiotropium, another LAMA, improves lung function and decreases asthma exacerbation [12, 37]. These findings support the notion that triple therapy with UMEC added to FF/VI may be effective in patients with ACO, as an entity of overlapping asthma and COPD. The FF administered in this study is an ICS having greater glucocorticoid receptor affinity, longer residency time in the human lung, and greater topical potency compared with other available ICS [38]. The beneficial features of FF may be suitable especially for severe asthma and COPD, in which high levels of oxidative stress are seen [38]. Moreover, VI provides up to 24 hours of fast-acting relief of the symptoms suffered by both asthma and COPD patients, through a rapid and prolonged action to improve lung function [39]. The findings described above provide a strong rationale for the use of UMEC added to FF/VI in patients with ACO to maximize clinical benefits and lung function and to thereby prevent exacerbation. 

This study has several limitations to be considered. Firstly, the results describe many statistically significant improvements in respiratory function tests; however, these results by themselves cannot express the etiology of the clinically beneficial effect. A potent bronchodilatory effect may be the main force behind the results of our study, but an examination of airway mediators would help to define this mechanism; the anti-inflammatory properties of LAMAs reportedly may contribute to this beneficial effect [34]. Further studies are needed to investigate the mechanisms underlying the bronchodilatory effects of triple therapy with UMEC added to FF/VI. Secondly, a specific, formal definition of ACO has yet to be determined [2, 15, 29]. We conducted this study using physiological lung function criteria described in previous studies [1, 2, 15, 20, 21, 29]. Other definitions have been proposed based on a specific inflammatory pattern [13], but these are not specific for asthma and COPD: for example, airway eosinophilia has been shown in only 48% of patients with asthma and 34% of those with COPD [2]. In contrast, concordance with the proportion of ACO patients in several different studies suggests the validity of the physiological lung function criteria [2, 15, 16, 17]. Nevertheless, further studies are required to establish a consensus and better diagnostic criteria for ACO. Thirdly, pulmonary physiology outcome measures confirm the bronchodilatory effect of LAMAs, but ACT scores and CAT scores did not show benefits with respect to healthcare outcome. We suspect that the number of patients enrolled in this study was insufficient to detect such an effect as this was a pilot study conducted without power calculation. This study was also relatively short for assessment of the difference between pulmonary physiology outcomes and patient reported outcomes. We speculate that the relatively short treatment period might result in carry-over of the beneficial effect of UMEC to the following period. A larger and longer study may be required to clarify the benefits of this treatment with respect to healthcare outcome. We hope that a larger study will demonstrate clinically important differences in symptoms rather than solely showing statistical significance in lung function as observed in the present pilot study. Finally, patients with ACO are considered to comprise two subtypes: asthmatic patients with persistent airflow limitation and COPD patients with a partial response to bronchodilatory therapy [2]. To date, these two conditions cannot be classified accurately. Further studies are required to address the phenotypes of ACO and their relationship to treatment choices. 

## Conclusion 

In conclusion, 4-week once-daily treatment of ACO patients with UMEC 62.5 µg added to FF/VI 200/25 µg produced significant improvements from baseline in lung function compared with once-daily treatment with FF/VI 200/25 µg alone, and with a comparable safety profile in this pilot study. These findings indicate that triple therapy with UMEC added to FF/VI has potential as a regular treatment for ACO. This is also the first report demonstrating the therapeutic effect of ICS/LABA/LAMA triple therapy in patients with ACO. Further large studies are required to investigate the effect of other ICS/LABA/LAMA combinations, and more interestingly, to define the precise etiology of their clinical efficacy. 

## Acknowledgment 

We thank Nicholas Rufaut, PhD, and Ryan Chastain-Gross, PhD from Edanz Group (www.edanzediting.com/ac) for editing a draft of this manuscript. 

## Funding 

No funding was related to this study. 

## Conflict of interest 

The authors declare that they have no competing interests. 

**Table 1.** Patient characteristics.

Age (years)	70.1 ± 9.0, range 54 – 87
Gender (male/female)	17/0
Body mass index (kg/m^2^)	21.5 ± 2.4
History of smoking (pack-years)	66.6 ± 39.5
Treatment of theophylline (with/without)	10/7
Treatment of carbocysteine (with/without)	10/7
Treatment of LTRA (with/without)	14/3
DL_CO_ as % predicted	69.8 ± 17.9
DL_CO_/VA as % predicted	46.2 ± 13.8
Bronchodilator response (%)*	18.8 ± 4.1

LTRA = leukotriene receptor antagonist; DL_CO_ = lung carbon monoxide diffusing capacity; VA = alveolar volume. *Bronchodilator response means percent increase in forced expiratory volume in 1 second (FEV_1_) from the baseline value inhalation of 200 µg of salbutamol sulfate. Data are presented as mean (± SD).

**Table 2.** Spirometry parameters after each treatment.

	Run-in	FF/VI	FF/VI/UMEC	p-value
Spirometry parameters				
VC (as % predicted)	82.8 (12.1)	86.4 (12.5)	91.8 (14.9)	< 0.01
FVC (as % predicted)	81.5 (15.2)	84.4 (15.9)	89.2 (16.1)	< 0.01
FEV_1_ (as % predicted)	46.8 (12.8)	49.3 (14.0)	54.4 (13.7)	< 0.01
FEV_1_/FVC (%)	46.2 (10.9)	49.3 (14.0)	54.4 (13.6)	< 0.01
PEF (as % predicted)	45.1 (16.1)	46.4 (15.5)	52.8 (15.5)	< 0.01
FEF_25–75%_ (as% predicted)	14.6 (5.6)	15.7 (7.9)	17.8 (7.4)	0.09
MEF_50_ (as % predicted)	16.4 (7.2)	17.4 (9.5)	20.4 (10.0)	< 0.05
MEF_25_ (as % predicted)	14.6 (5.6)	15.7 (7.9)	17.8 (7.4)	0.09

FP/SAL = fluticasone propionate/salmeterol; FFM = fulticasone furoate/vilanterol; VC = vital capacity; FVC = forced vital capacity; FEV_1_ = forced expiratory volume in 1 second; PEF = peak expiratory flow; FEF_25–75%_ = forced expiratory flow at 25 – 75%; MEF_50_ = maximum expiratory flow rate at 50% forced vital capacity; MEF_25_ = maximum expiratory flow rate at 25% forced vital capacity. Data are presented as mean (SD).

**Table 3.** FOT parameters, FeNO, CAT, ACT, and blood examination parameters after each treatment.

	Run-in	FF/VI	FF/VI/UMEC	p-value
FOT parameters				
R5 (cmH_2_O/L/s)	3.6 (0.9)	3.6 (0.9)	3.1 (0.9)	< 0.05
R20 (cmH_2_O/L/s)	2.6 (0.5)	2.6 (0.4)	2.3 (0.6)	0.24
R5-20 (cmH_2_O/L/s)	1.1 (0.5)	1.0 (0.6)	0.7 (0.5)	< 0.01
X5 (cmH_2_O/L/s)	–1.6 (1.0)	–1.5 (1.0)	–1.0 (0.8)	< 0.01
Fres (Hz)	17.0 (4.7)	15.6 (5.7)	13.0 (5.8)	< 0.01
ALX (cmH_2_O/L/s × Hz)	12.4 (9.5)	11.2 (10.5)	6.9 (7.8)	< 0.01
FeNO (ppb)	13.3 (11.8)	13.2 (11.4)	11.4 (10.8)	0.33
COPD Assessment Test score	15.1 (6.1)	13.8 (7.3)	13.4 (6.1)	0.18
Asthma Control Test score	22.5 (7.0)	22.1 (5.4)	22.9 (7.5)	0.78
Serum total IgE (IU/mL)	420.6 (649.6)	380.8 (556.1)	408.1 (621.6)	0.38
Peripheral eosinophil count (/µL)	227.5 (140.8)	222.2 (139.3)	236.6 (201.7)	0.78

FOT = forced oscillation tequnique; R5 and R20 = respiratory system resistance at 5 Hz and 20 Hz; X5 = respiratory system reactance at 5 Hz; Fres = resonant frequency; ALX = area of low reactance; FeNO = the fraction of exhaled nitric oxide; COPD = chronic obstructive pulmonary disease; Ig = immunoglobrin. Data are presented as mean (SD). One-way analysis of variance (ANOVA) was used for analyzing differences in respiratory functions.
